# Development of a Low-Cost Artificial Vision System as an Alternative for the Automatic Classification of Persian Lemon: Prototype Test Simulation

**DOI:** 10.3390/foods12203829

**Published:** 2023-10-19

**Authors:** Bridget V. Granados-Vega, Carlos Maldonado-Flores, Camila S. Gómez-Navarro, Walter M. Warren-Vega, Armando Campos-Rodríguez, Luis A. Romero-Cano

**Affiliations:** 1 Grupo de Investigación en Materiales y Fenómenos de Superficie, Departamento de Biotecnológicas y Ambientales, Universidad Autónoma de Guadalajara, Av. Patria 1201, Zapopan 45129, Mexico; bridget.granados@edu.uag.mx (B.V.G.-V.); camila.gomez@edu.uag.mx (C.S.G.-N.); wwarrenv12@gmail.com (W.M.W.-V.); 2 Laboratorio de Innovación y Desarrollo de Procesos Industriales Sostenibles, Departamento de Biotecnológicas y Ambientales, Universidad Autónoma de Guadalajara, Av. Patria 1201, Zapopan 45129, Mexico; carlos.maldonado@edu.uag.mx

**Keywords:** MATLAB Simulink model prototype, quality control, colorimetric chart, artificial neural networks, electronic eye

## Abstract

In the present research work, an algorithm of artificial neural network (ANN) has been developed based on the processing of digital images of Persian lemons with the aim of optimizing the quality control of the product. For this purpose, the physical properties (weight, thickness of the peel, diameter, length, and color) of 90 lemons selected from the company Esperanza de San José Ornelas SPR de RL (Jalisco, Mexico) were studied, which were divided into three groups (Category “extra”, Category I, and Category II) according to their characteristics. The parameters of weight (26.50 ± 3.00 g), diameter/length (0.92 ± 0.08) and thickness of the peel (1.50 ± 0.29 mm) did not present significant differences between groups. On the other hand, the color (determined by the RGB and HSV models) presents statistically significant changes between groups. Due to the above, the proposed ANN correctly classifies 96.60% of the data obtained for each of the groups studied. Once the ANN was trained, its application was tested in an automatic classification process. For this purpose, a prototype based on the operation of a stepper motor was simulated using Simulink from Matlab, which is connected to three ideal switches powered by three variable pulse generators that receive the information from an ANN and provide the corresponding signal for the motor to turn to a specific position. Manual classification is a process that requires expert personnel and is prone to human error. The scientific development presented shows an alternative for the automation of the process using low-cost computational tools as a potential alternative.

## 1. Introduction

In Mexico, two varieties of lemon are mainly cultivated, the Persian and the Mexican, corresponding to 30% and 70% of the total production. The Persian lemon is dedicated almost exclusively to export, so its quality controls are strict to satisfy the demands of the market [1]. According to information from national producers, lemons for export can be classified according to their characteristics in: (i) Category “extra”, which must have a superior quality and must not present defects, except for very slight superficial ones as long as the general appearance of the product is not affected; (ii) Category I, which has slight defects in shape, color, and surface that do not exceed 1 cm^2^; and (iii) Category II, those lemons that do not fit the previously mentioned categories and that present superficial defects that exceed 1 cm^2^. Once the lemon is harvested, most farmers carry out this classification manually, which generates errors in the quality of the process, making it essential to use an artificial vision system that allows the process to be automated and avoid human errors [2].

In this sense, recent research has focused on proposing solutions based on the use of artificial vision and machine learning to solve this type of problem [3]. Through the use of artificial intelligence, such as the implementation of artificial neural networks, it has been possible to evaluate complex systems for the prediction and control of different areas in the food industry, such as extrusion [4], process conditions (formulations) [5], drying [6], or evaluating its functional content [7]. In the last few years, artificial neural networks have proven to be advantageous because they allow the evaluation of complex and multivariable systems, thus providing an indicator that demonstrates the quality of a product. For example, the analysis to estimate total chlorophyll content in apples has been studied by color analysis and spectra data [8], or measuring six color values from surface images to predict texture characteristics such as hardness and gumminess [9]. Another use of artificial neural networks is to classify products by their physicochemical properties and be discriminant with respect to location, composition, or quality [10,11,12]. In this sense, various studies have been reported for the evaluation of the quality of fruits using image analysis in combination with convolutional models of neural networks (CNN), among which studies in apples [13], lemons [14], grapevine [15], papaya [16], tomato [17], strawberry [18], blueberry [19], banana [20], and melon [21] stand out. In all cases, the studies are based on the acquisition of data using digital cameras, which are subsequently processed mathematically to obtain the RGB (RGB color model is an additive color model in which the red, green, and blue primary colors of light are added together in various ways to reproduce a broad array of colors) and/or HSV (the HSV model is an alternative representation of the RGB color model in which the hue, saturation, and value are represented) values in order to provide this information to the designed CNN algorithm. The data set obtained is entered into the network for training to obtain a result. After the analysis, the error between the given labels and the output predictions is calculated using a loss function to propagate the error throughout the network by means of a backpropagation algorithm. Finally, the weights are updated to minimize the error, and the procedure is repeated until it converges or reaches a limit of iterations [22].

However, although there is now a lot of information reported on the use of artificial intelligence to evaluate the quality of different fruits, it must be integrated into automation systems to achieve product control at an industrial level. In the food industry, the automation of processes becomes essential since its selection is more efficient than human work due to the minimization of errors [23,24,25,26]. Based on this, the objective of this research corresponds to the development of a prototype and its potential application in the automatic classification of the three categories that define the quality of a Persian lemon. To achieve these, an artificial neural network was developed and trained using the physicochemical properties of the fruit for subsequent programming in the prototype model and testing its operation in a computer-assisted simulation.

## 2. Materials and Methods

### 2.1. Samples

**Figure 1:**
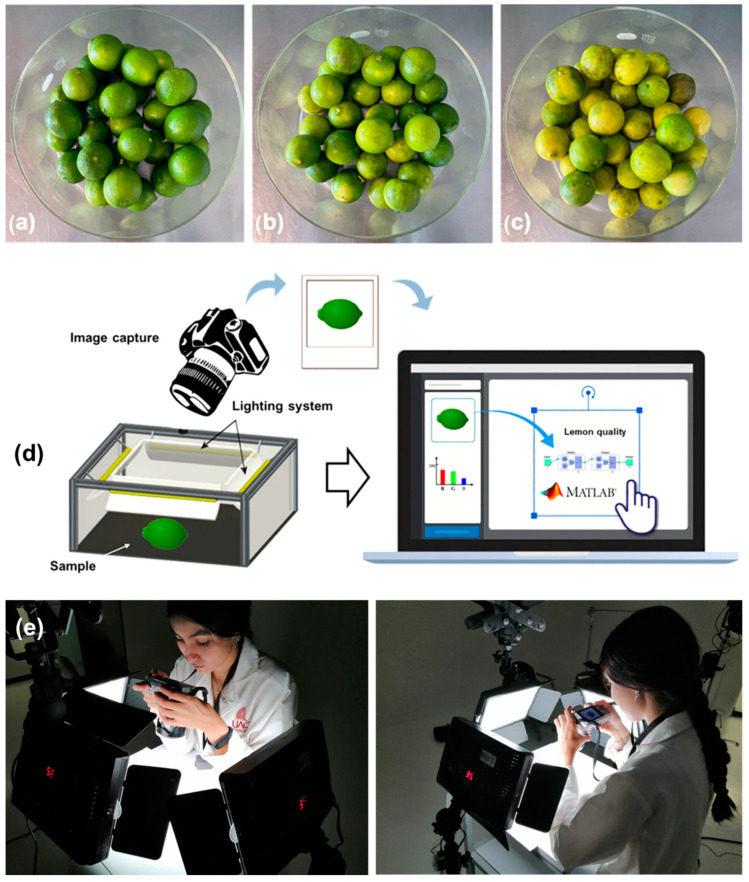
Photographs of the visual classification of lemons: (**a**) Category “extra”, (**b**) Category I, and (**c**) Category II. (**d**) Visual artificial system to classify lemons; (**e**) experimental Set-up.

A batch of 90 Persian lemons (*Citrus* × *latifolia*) was selected from the company Esperanza de San José Ornelas SPR de RL (Jalisco, Mexico), which is dedicated to the production and distribution of various fruits (blueberry, corn, and Persian lemon). The samples were harvested and immediately classified by the expert workers of the company, who have more than 20 years of experience; then, within 1 day, the lemons were transported to our laboratory. Of these, 30 correspond to the Category “extra”, 30 to Category I, and 30 to Category II (Figure 1).

STATISTICA 10.0 software (StatSoft, Palo Alto, CA, USA) was used to analyze the experimental data. The descriptive statistics analysis was performed by determining the mean and standard deviation. Additionally, a one-way analysis of variance (ANOVA) was employed to determine the existence of statistically significant differences between the means of the groups (Category extra, Category I, and Category II) using a significance level of 95%.

### 2.2. Color Study Using Digital Image Processing

The digital image processing was carried out in a similar way as previously reported by Warren-Vega et al. [27]. It is important to highlight that one of the limitations of the study is related to the fact that the classification of fruits based on image processing is sensitive to the lighting conditions and the background color at the time of taking the photograph, so it is important to standardize the method. Due to the above, digital images were performed under controlled light conditions and were subjected to continuous and direct illumination from a white LED light source (45 W) at a power of 35%. All photos were taken at a distance of 30 cm between the object and the camera. Each sample was analyzed in duplicate, performing 3 repetitions to ensure the repeatability and reproducibility of the measures. The operating conditions of the camera were taken with the manual option, at a lens objective of 18–25 mm and a lens aperture in a range of 4 to 5 at an ISO 500. (Note: camera specifications do not limit the application of the method. However, the size of the photography area selected for RGB decomposition needs to be observed to contain a minimum number of pixels to provide the same analytical sensitivity). Using its CCD (Charge-Couple Device, Sony Corp., Bangkok, Thailand) detector, it is possible to capture digital images and convert them into a voltage sequence that can be translated into an analytical signal. The digital images obtained for each sample were decomposed using the RGB model (red, green, and blue), and Color Intensity (C.I.) was obtained from the sum of the three values (C.I. = red + green + Blue).

Image Analysis. The digital images obtained were saved as individual files in jpeg format. The average size per image was 3 MB (14.2 megapixel resolution, 4592 × 3056 pixels). Image processing consisted of selecting and clipping a region of interest (defined as at least 60% of the total area of the fruit resulting in a new image with a dimension of 843 × 880 pixels. The obtained images were decomposed according to the RGB color model into distribution histograms for each channel (red, green, and blue) using the MATLAB R2023a software. The results obtained were corroborated using the free app Color Name^®^ (version 3.2, 2022). After that, the values of RGB were transformed to HSV (Hue, Saturation, and Value), which resembles color in human perception [28].

### 2.3. Physical Properties Analysis in Lemons

Lemon measurements were performed to obtain the following characteristics: thickness, weight, length, and diameter. For measurements in lemons, a NIMBUS analytical balance and a digital Vernier were employed.

### 2.4. Artificial Neural Network Approach

**Figure 2:**
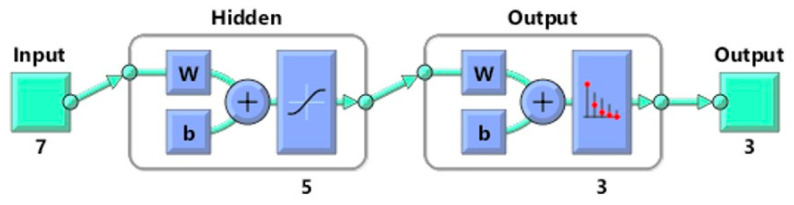
ANN architecture for lemon classification.

The MATLAB R2023a program was used to establish an artificial neural network algorithm that allows the classification of lemons based on quality standards (Category “extra”, Category I, and Category II). In the case of the inputs, physical properties and colorimetric measurements (intensity of color, hue, saturation, and value) were used as classifiers to obtain the group to which they belonged (outputs). The optimal conditions for the artificial neural network architecture consisted of a total of 5 neurons with a sigmoid hidden layer and a softmax output layer to permit a correct classification. The network will be trained using the scaled conjugated gradient method. To evaluate the performance of the ANN, cross-entropy performance was used to establish the best architecture. The training of the neural network consisted as follows: 60 lemons (n = 20 lemons from each group) were used to obtain the algorithm, in which data was randomly divided into training (70%), validation (15%), and testing (15%). Subsequently, the remaining 30 lemons were used as a second set with the objective of establishing their effectiveness. The architecture of the neural network is shown in Figure 2.

### 2.5. MATLAB Simulink Model Prototype

A model prototype was developed to evaluate the feasibility of the automatic classification of lemons using the proposed ANN. For this purpose, input and output interfaces were developed using control algorithms in Simulink. The proposed model consisted of connecting the ANN to three variable pulse generators, which, depending on the value returned by the ANN, activate an ideal switch connected to a stepper motor (motor type: variable reluctance, number of phases: 3, maximum winding inductance: 0.01, minimum winding inductance: 0.002, winding resistance 1.2 ohm, step angle: 120°, initial position: 0°, total inertia: 2 × 10^−5^ kg m^2^, and total friction: 0.001 N m), which rotates at 0°, 120° (clockwise) or −120° (anticlockwise) depending on the category of lemon detected by the ANN. It is proposed that turning the motor will send the lemon to the corresponding basket according to the corresponding category.

## 3. Results and Discussion

### 3.1. Physicochemical Parameters of Persian Lemons: Analysis of Categories

**Figure 3:**
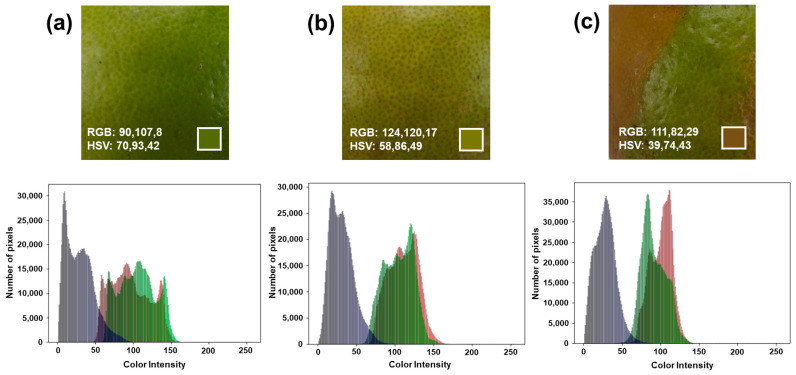
Representative sample of digital images and RGB color distribution for each of the categories of lemons analyzed: (**a**) Category extra, (**b**) Category I, and (**c**) Category II.

Within the three different groups of lemons analyzed based on their quality, the RGB analysis was carried out to appreciate differences in the colors presented by the fruits. Figure 3 and Supplementary Figures S1–S3 present representative samples of the three categories analyzed. At least 60% of the total fruit area was studied in all cases. Once the digital images were obtained, they were decomposed into the primary colors red, green, and blue. The results are presented as an RGB color histogram, representing its distribution in the image. From this information, the maximum values of each of the analyzed colors were defined. For the category extra, the green color predominates (R = 90, G = 107, B = 8) and is characteristic of the fruit freshly harvested.

On the other hand, for categories I and II, there is a displacement of the red color, almost wholly overlapping with the green color in category I (R = 124, G = 120, B = 17), resulting in a yellow coloration characteristic of fruit ripening. Finally, category II stands out for the superposition of red and green color with a majority tendency toward the intensity of the red tone (R = 111, G = 82, B = 29), associated with the localized brown tone of the fruit caused by mechanical damage at harvest time. From this information, it is concluded that the area analyzed is representative of each of the fruits studied, evidencing that the proposed method satisfactorily identifies minor alterations.

Using the RGB analysis obtained from the decomposition of the images, the color intensity value was calculated as the first variable for the artificial neural network.

**Figure 4:**
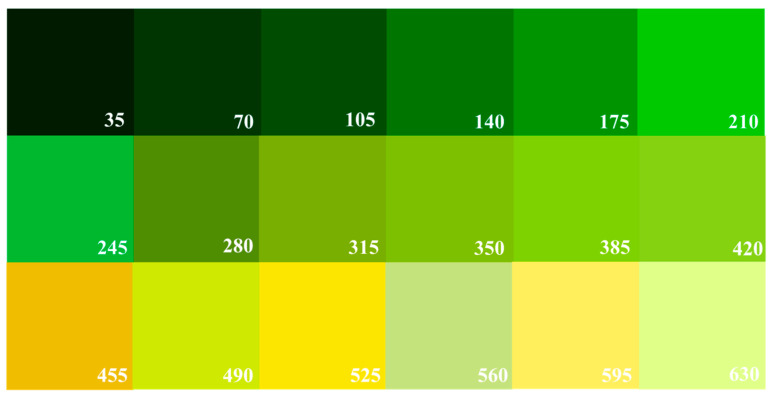
Color chart of the lemons analyzed based on their quality criteria. The number corresponds to Color Intensity (CI). From top to bottom: Category “extra” CI (35–210), Category I CI (245–420), and Category II CI (455–630).

To demonstrate the color differences presented in the groups, Figure 4 was made to demonstrate the variability that the camera sensor detected and how it was perceived. In the color chart, the extra category of lemons is mainly green, because that is the way consumers prefer it in North America. At this stage, the fruit has not yet reached maturity, so the main pigment in the rind corresponds to chlorophyll. As the fruit begins to ripen, its color tones change from light green tones (category I) to yellow tones (category II), in which the pigment of the rind is mainly carotenoids [29]. For this reason, the changes in the color of the fruit are related to its maturity, in which the degradation of chlorophylls occurs in four stages: (i) synthesis of Mg-protoporphyrin IX from glutamic acid through a series of reactions and then conversion to chlorophyll a; (ii) interconversion of chlorophyll a and chlorophyll b, also known as the chlorine leaf plant cycle; (iii) chlorophyll binding; and (iv) degradation of chlorophyll [30].

**Figure 5:**
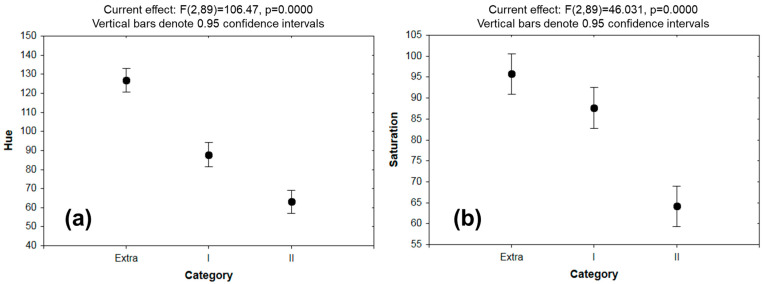

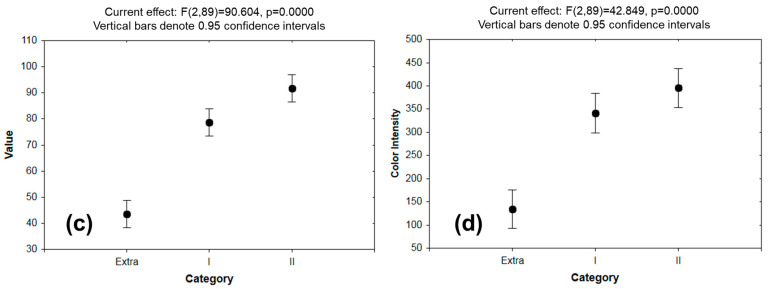
Statistical analysis of the values of (**a**) hue, (**b**) saturation, (**c**) value, and (**d**) color intensity in Persian lemons. Where: Category “extra” = high quality, Category I = medium quality, and Category II = low quality.

Finally, the RGB data was converted to another color space, HSV, which has demonstrated better results in evaluating fruit properties by machine learning methods [29]. In Figure 5 and Supplementary Figure S4, the statistical analysis of hue, saturation, value, color intensity, red, green, and blue can be appreciated. Saturation was the only property that demonstrated a not-significant difference between the excellent and medium groups. With this information, it is concluded that there are differences in the colors, thus establishing significant variables for the design of the ANN architecture, as has been reported by other authors [31,32].

To complete the characterization of the lemons studied, the properties of weight, sphericity (Diameter/Length, D/L) and thickness of the peel were evaluated. The results are presented in Table 1 and Supplementary Figure S5. It is observed that there are no significant differences between the properties studied in the categories of fruit quality (weight *p*-value = 0.00017 < 0.05; D/L *p*-value = 0.181327 > 0.05; thickness *p*-value = 0.22179 > 0.05). For all categories, the weight was 25 g with a peel thickness of 1.5 mm on average, resulting in spherical fruits (D/L = 0.9). The foregoing shows that the consumer’s perception of the quality of the Persian lemon depends exclusively on the maturity of the fruit, which can be measured from image analysis.

From this information, the physicochemical parameters of lemons are going to be used to feed the ANN for Persian lemon classification.

### 3.2. Construction and Training of the Proposed ANN

**Figure 6:**
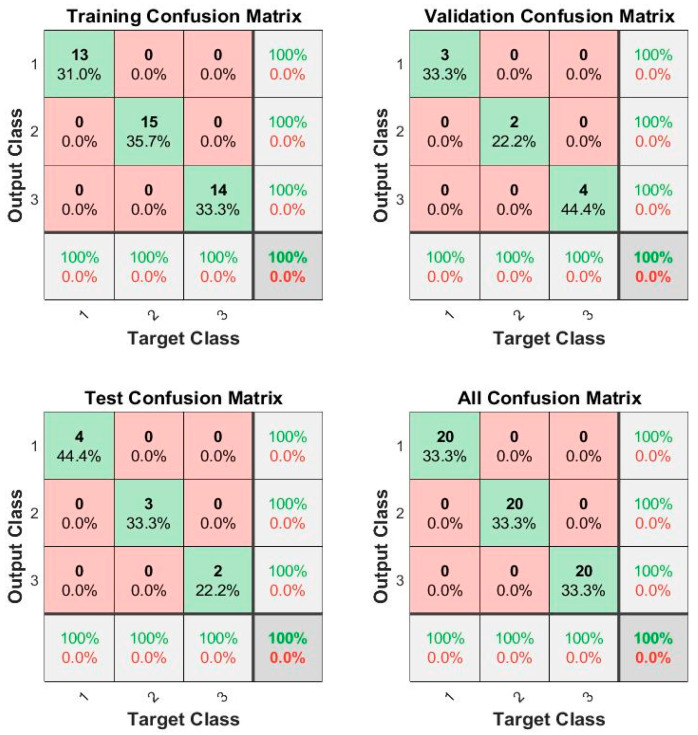
Confusion matrix for lemon classification (outputs: 1: Category “extra”, 2: Category I, 3: Category II).

Having this, the neural network was used to classify between the different groups, having within the training a 100% classification within the confusion matrix using 60 lemons, as shown in Figure 6.

**Figure 7:**
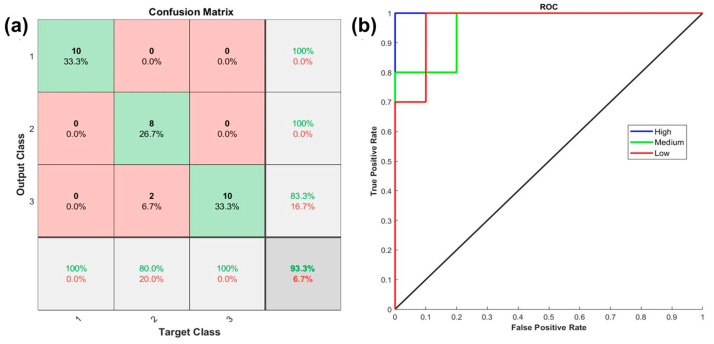
ANN second set (**a**) confusion matrix and (**b**) ROC curve.

To evaluate the feasibility of the ANN, it was proposed to perform a second set, in which the remaining 30 lemons were employed to verify that the algorithm would allow classifying future cases. The results are shown in Figure 7. It can be appreciated that a 93.3% correct classification could be achieved with two misclassified lemons as Category II being Category I. In the case of the ROC curve, it can be appreciated that the true positive rate is 1 for Category “extra”, then 0.8 for Category I, and finally 0.7 for Category II.

With the result of both validations, a value of 96.60% of the correct classification is obtained using the physical and colorimetric properties of the analyzed fruits.

The results obtained show that the use of artificial neural networks for the classification of lemon quality is feasible. Similar results have been previously reported showing that the use of machine learning is helpful for fruit classification (Table 2); however, in all cases, the studies focus on the development of basic principles, which, according to the Technology Readiness Level (TRL), classify the technology at level 1 (TRL1). In order to show an advance with respect to the previously reported studies, proofs of concept were carried out by connecting the proposed artificial neural networks to a stepper motor (Section 3.3) to demonstrate the usefulness of the technology in developing an automatic classification system at an industrial level. Based on the TRL, these results classify the proposed technology at a TRL3 level.

### 3.3. MATLAB Simulink Model Prototype: Test Simulation

**Figure 8:**
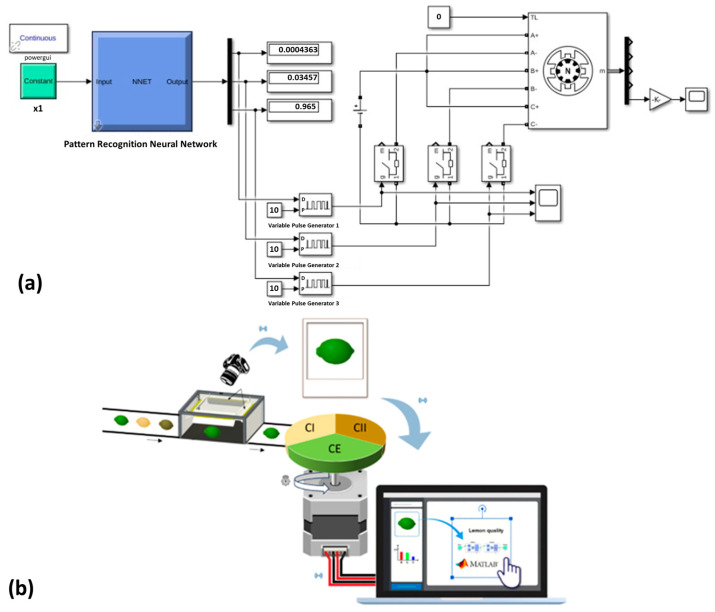
(**a**) MATLAB Simulink model prototype; (**b**) proposed diagram for the automatic classification of lemons.

**Figure 9:**
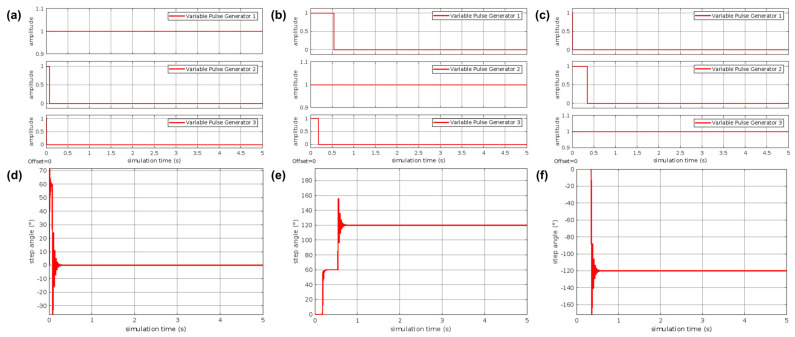
Pulse outputs (**a**–**c**) and step angles (**d**–**f**) with different delay times based on the classification of lemons using the trained ANN.

In order to show the technological maturity of the proposed prototype, the simulation of the device was carried out using the Simulink model presented in Figure 8. The simulation results are shown in Figure 9.

Figure 9a–c shows typical forms of amplitude modulated pulse output at 0 and 1 corresponding to the input duty cycle by changing the on/off delay time of the switches. It is observed that depending on the signal sent by the ANN, only one of the three pulse generators gives an amplitude equal to 1, which is translated into the rotation of the stepper motor (Figure 9d–f) depending on the category of lemon detected. It should be noted that the turn response time is dependent on the probability value thrown by the ANN; that is, for values close to 1.0, the turn response is fast and decreases as the value of the probability of success of the ANN decreases. However, in none of the cases tested, the response time was greater than 1 s, so the proposed prototype is feasible for use.

Based on the above, the system shown in Figure 7b is proposed as a prototype of an automatic lemon quality classifier, which consists of taking a photograph that is decomposed according to the RGB and HSV models. This information is sent to the MATLAB software in which the previously trained ANN is used, and the resulting information is sent to the controller (stepper motor), which transforms the digital signal into a mechanical rotation of the axis capable of diverting the lemon to the corresponding basket according to your category. From the results obtained, it is concluded that the technological maturity of the proposed device is at a Technology Readiness Level (TRL) of 3 since a proof-of-concept has been satisfactorily evaluated.

## 4. Conclusions

The present study shows the basis for the development of a simple, low-cost, and fast technology for the automatic classification of Persian lemons according to their quality parameters to preserve the standards requested by the market. It is possible to use a low-cost device to analyze digital images and, coupled with an ANN, make decisions to differentiate between categories. The algorithm developed to analyze digital images shows 96.6% accuracy for product classification, showing itself as a potential application to automate manual fruit classification processes, helping to reduce errors made by personnel and increasing the speed of the process with the aim of having low costs. The proposed prototype model has been evaluated through simulations, evidencing its feasibility of use. As a perspective, it is contemplated to evaluate the proposed prototype on a pilot scale under controlled conditions in order to show the proposal with a level of technological maturity of TRL4 (technology validated in the lab).

## Figures and Tables

**Table 1:** Input variables in an artificial neural network for the classification of lemons.

Properties	Category “Extra”	Category I	Category II
Hue	126.83 ± 7.23	87.83 ± 25.60	60.95 ± 9.08
Saturation	95.60 ± 7.21	87.85 ± 11.07	62.44 ± 17.36
Value	42.39 ± 20.85	78.65 ± 12.05	91.84 ± 4.88
Color intensity	127.43 ± 56.62	341.2 ± 68.54	538.55 ± 57.49
Weight (g)	26.50 ± 3.00	25.69 ± 3.02	23.05 ± 3.08
Diameter/Length	0.92 ± 0.08	0.91 ± 0.06	0.97 ± 0.07
Thickness (mm)	1.50 ± 0.29	1.48 ± 0.32	1.29 ± 0.33

**Table 2:** Bibliographic comparison against outstanding works previously reported in the literature.

Fruit	Study	Goal	Ref.
Apple	Laser-induced light backscattering imaging and convolutional neural network	Automatic identification and detection of apple defects achieving a recognition rate of 92.5%	[13]
Lemon	Stochastic Pooling Mechanism in Deep Convolutional Neural Networks	Detect defects and grade them achieving a recognition rate of 100%	[14]
Papaya	CNNs * and Convolutional Block Attention Modules	disease detector with a mean average precision of 86.2%	[33]
Tomato	Deep CNN *	nutritional deficiencies detection in the cropping process with a mean average precision of 91%	[17]
Strawberry	Deep CNN *	quality classification achieving a recognition rate of 96.49%	[18]
Blueberry	CNN *, ResNet *, ResNeXt *	Detection of internal mechanical damage achieving a recognition rate of 89.5%	[19]
Banana	CNN *	Fine-grained classification of banana’s ripening stages achieving a recognition rate of 94.4%	[20]
Melon	CNN *	Skin lesion achieving an accuracy rate of 98.5%	[21]

* CNN—Convultional Neural Network; ResNet—Residual Network; ResNeXt—Improved version of ResNet.

## Data Availability

The data that support the findings of this study are available from the corresponding author upon reasonable request.

## Supplementary Materials

The following supporting information can be downloaded at: https://www.mdpi.com/article/10.3390/foods12203829/s1. Supplementary Figure S1. Representative sample of 10 lemons analyzed for category “extra”. Note: The differences in the dimensions of the images correspond to the condition of analyzing at least 60% of the total area of the fruit. Supplementary Figure S2. Representative sample of 10 lemons analyzed for category I. Note: The differences in the dimensions of the images correspond to the condition of analyzing at least 60% of the total area of the fruit. Supplementary Figure S3. Representative sample of 10 lemons analyzed for category II. Note: The differences in the dimensions of the images correspond to the condition of analyzing at least 60% of the total area of the fruit. Supplementary Figure S4. Statistical analysis of the values of (a) red, (b) green, and (c) blue in Persian lemons according to the category. Supplementary Figure S5. Statistical analysis of the values of (a) weight, (b) diameter/length, and (c) thickness in Persian lemons according to the category.

## References

[1] Raddatz-Mota D., Franco-Mora O., Mendoza-Espinoza J.A., Rodríguez-Verástegui L.L., Díaz de León-Sánchez F., Rivera-Cabrera F. (2019). Effect of Different Rootstocks on Persian Lime (*Citrus latifolia* T.) Postharvest Quality. Sci. Hortic..

[2] Mukhtar T., Jamil S., Arif U., Razzaq W., Wasif M. (2021). Lemon Grading and Sorting Using Computer Vision. Eng. Proc..

[3] Nayak J., Vakula K., Dinesh P., Naik B., Pelusi D. (2020). Intelligent Food Processing: Journey from Artificial Neural Network to Deep Learning. Comput. Sci. Rev..

[4] Cubeddu A., Rauh C., Delgado A. (2014). Hybrid Artificial Neural Network for Prediction and Control of Process Variables in Food Extrusion. Innov. Food Sci. Emerg. Technol..

[5] Batista L.F., Marques C.S., Pires A.C.d.S., Minim L.A., Soares N.d.F.F., Vidigal M.C.T.R. (2021). Artificial Neural Networks Modeling of Non-Fat Yogurt Texture Properties: Effect of Process Conditions and Food Composition. Food Bioprod. Process..

[6] Zalpouri R., Singh M., Kaur P., Kaur A., Gaikwad K.K., Singh A. (2023). Drying Kinetics, Physicochemical and Thermal Analysis of Onion Puree Dried Using a Refractance Window Dryer. Processes.

[7] da Silva Sauthier M.C., da Silva E.G.P., da Silva Santos B.R., Silva E.F.R., da Cruz Caldas J., Cavalcante Minho L.A., dos Santos A.M.P., dos Santos W.N.L. (2019). Screening of *Mangifera indica* L. Functional Content Using PCA and Neural Networks (ANN). Food Chem..

[8] Pourdarbani R., Sabzi S., Hernández-Hernández M., Hernández-Hernández J.L., Gallardo-Bernal I., Herrera-Miranda I. (2020). Non-Destructive Estimation of Total Chlorophyll Content of Apple Fruit Based on Color Feature, Spectral Data and the Most Effective Wavelengths Using Hybrid Artificial Neural Network—Imperialist Competitive Algorithm. Plants.

[9] Fan F.H., Ma Q., Ge J., Peng Q.Y., Riley W.W., Tang S.Z. (2013). Prediction of Texture Characteristics from Extrusion Food Surface Images Using a Computer Vision System and Artificial Neural Networks. J. Food Eng..

[10] Debska B., Guzowska-Swider B. (2011). Application of Artificial Neural Network in Food Classification. Anal. Chim. Acta.

[11] Cimpoiu C., Cristea V.M., Hosu A., Sandru M., Seserman L. (2011). Antioxidant Activity Prediction and Classification of Some Teas Using Artificial Neural Networks. Food Chem..

[12] da Silva C.E.T., Filardi V.L., Pepe I.M., Chaves M.A., Santos C.M.S. (2015). Classification of Food Vegetable Oils by Fluorimetry and Artificial Neural Networks. Food Control.

[13] Wu A., Zhu J., Ren T. (2020). Detection of Apple Defect Using Laser-Induced Light Backscattering Imaging and Convolutional Neural Network. Comput. Electr. Eng..

[14] Jahanbakhshi A., Momeny M., Mahmoudi M., Zhang Y.D. (2020). Classification of Sour Lemons Based on Apparent Defects Using Stochastic Pooling Mechanism in Deep Convolutional Neural Networks. Sci. Hortic..

[15] Barré P., Herzog K., Höfle R., Hullin M.B., Töpfer R., Steinhage V. (2019). Automated Phenotyping of Epicuticular Waxes of Grapevine Berries Using Light Separation and Convolutional Neural Networks. Comput. Electron. Agric..

[16] Munasingha L.V., Gunasinghe H.N., Dhanapala W.W.G.D.S. Identification of Papaya Fruit Diseases Using Deep Learning Approach. Proceedings of the 4th International Conference on Advances in Computing and Technology (ICACT–2019).

[17] Tran T.T., Choi J.W., Le T.T.H., Kim J.W. (2019). A Comparative Study of Deep CNN in Forecasting and Classifying the Macronutrient Deficiencies on Development of Tomato Plant. Appl. Sci..

[18] Sustika R., Subekti A., Pardede H.F., Suryawati E., Mahendra O., Yuwana S. (2018). Evaluation of Deep Convolutional Neural Network Architectures for Strawberry Quality Inspection. Int. J. Eng. Technol..

[19] Wang Z., Hu M., Zhai G. (2018). Application of Deep Learning Architectures for Accurate and Rapid Detection of Internal Mechanical Damage of Blueberry Using Hyperspectral Transmittance Data. Sensors.

[20] Zhang Y., Lian J., Fan M., Zheng Y. (2018). Deep Indicator for Fine-Grained Classification of Banana’s Ripening Stages. EURASIP J. Image Video Process.

[21] Tan W., Zhao C., Wu H. (2016). Intelligent Alerting for Fruit-Melon Lesion Image Based on Momentum Deep Learning. Multimed. Tools Appl..

[22] Naranjo-Torres J., Mora M., Hernández-García R., Barrientos R.J., Fredes C., Valenzuela A. (2020). A Review of Convolutional Neural Network Applied to Fruit Image Processing. Appl. Sci..

[23] Pandey V.K., Srivastava S., Dash K.K., Singh R., Mukarram S.A., Kovács B., Harsányi E. (2023). Machine Learning Algorithms and Fundamentals as Emerging Safety Tools in Preservation of Fruits and Vegetables: A Review. Processes.

[24] Zhu L., Spachos P., Pensini E., Plataniotis K.N. (2021). Deep Learning and Machine Vision for Food Processing: A Survey. Curr. Res. Food Sci..

[25] Zhang Y., Deng L., Zhu H., Wang W., Ren Z., Zhou Q., Lu S., Sun S., Zhu Z., Gorriz J.M. (2023). Deep Learning in Food Category Recognition. Inf. Fusion.

[26] Kakani V., Nguyen V.H., Kumar B.P., Kim H., Pasupuleti V.R. (2020). A Critical Review on Computer Vision and Artificial Intelligence in Food Industry. J. Agric. Food Res..

[27] Warren-Vega W.M., Contreras-Atrisco Z.A., Ramírez-Quezada M.F., Romero-Cano L.A. (2023). A Novel Approach of Artificial Intelligence for the Study of the Relation of Physicochemical Profile and Color Acquired by Tequila 100% Agave in Its Maturation Process. J. Food Compos. Anal..

[28] Heil J., Marschner B., Stumpe B. (2020). Digital Photography as a Tool for Microscale Mapping of Soil Organic Carbon and Iron Oxides. Catena.

[29] Rodrigo M.J., Alquézar B., Alós E., Lado J., Zacarías L. (2013). Biochemical Bases and Molecular Regulation of Pigmentation in the Peel of Citrus Fruit. Sci. Hortic..

[30] Tanaka A., Tanaka R. (2006). Chlorophyll Metabolism. Curr. Opin. Plant Biol..

[31] Cho B.H., Koseki S. (2021). Determination of Banana Quality Indices during the Ripening Process at Different Temperatures Using Smartphone Images and an Artificial Neural Network. Sci. Hortic..

[32] Mukherjee A., Sarkar T., Chatterjee K., Lahiri D., Nag M., Rebezov M., Shariati M.A., Miftakhutdinov A., Lorenzo J.M. (2022). Development of Artificial Vision System for Quality Assessment of Oyster Mushrooms. Food Anal. Methods.

[33] de Moraes J.L., de Oliveira Neto J., Badue C., Oliveira-Santos T., de Souza A.F. (2023). Yolo-Papaya: A Papaya Fruit Disease Detector and Classifier Using CNNs and Convolutional Block Attention Modules. Electronics.

