# Supplementary material for: Development of a Low-Cost Artificial Vision System as an Alternative for the Automatic Classification of Persian Lemon: Prototype Test Simulation
Source: Foods. 2023 Oct 19;12(20):3829. doi: 10.3390/foods12203829 (PMC10606287; doi:10.3390/foods12203829)
Supplement: Supplementary file 1 [file foods-12-03829-s001.zip › foods-2620883-supplementary.pdf]

foods

MDPI

Research Article

Development of a low-cost artificial vision system as an alternative for the automatic classification of Persian lemon: Prototype test simulation.

Bridget V. Granados-Vega [1], Carlos Maldonado-Flores [2], Camila S. Gómez-Navarro [1], Walter M. Warren-Vega [1], Armando Campos-Rodríguez [2,*,†], Luis A. Romero-Cano [1,*,†]

[1] Grupo de Investigación en Materiales y Fenómenos de Superficie. Departamento de Biotecnológicas y Ambientales. Universidad Autónoma de Guadalajara. Av. Patria 1201, C.P. 45129. Zapopan, Jalisco. México.

[2] Laboratorio de Innovación y Desarrollo de Procesos Industriales Sostenibles. Departamento de Biotecnológicas y Ambientales. Universidad Autónoma de Guadalajara. Av. Patria 1201, C. P. 45129. Zapopan, Jalisco. México

* Correspondence: armando.crodriguez@edu.uag.mx (A.C.-R.), luis.cano@edu.uag.mx (L.A.R-C.)

SUPPLEMENTARY MATERIAL

Citation: To be added by editorial staff during production.

Academic Editor: Firstname Lastname

Received: date
Revised: date
Accepted: date
Published: date

Copyright: © 2023 by the authors. Submitted for possible open access publication under the terms and conditions of the Creative Commons Attribution (CC BY) license (https://creativecommons.org/licenses/by/4.0/).

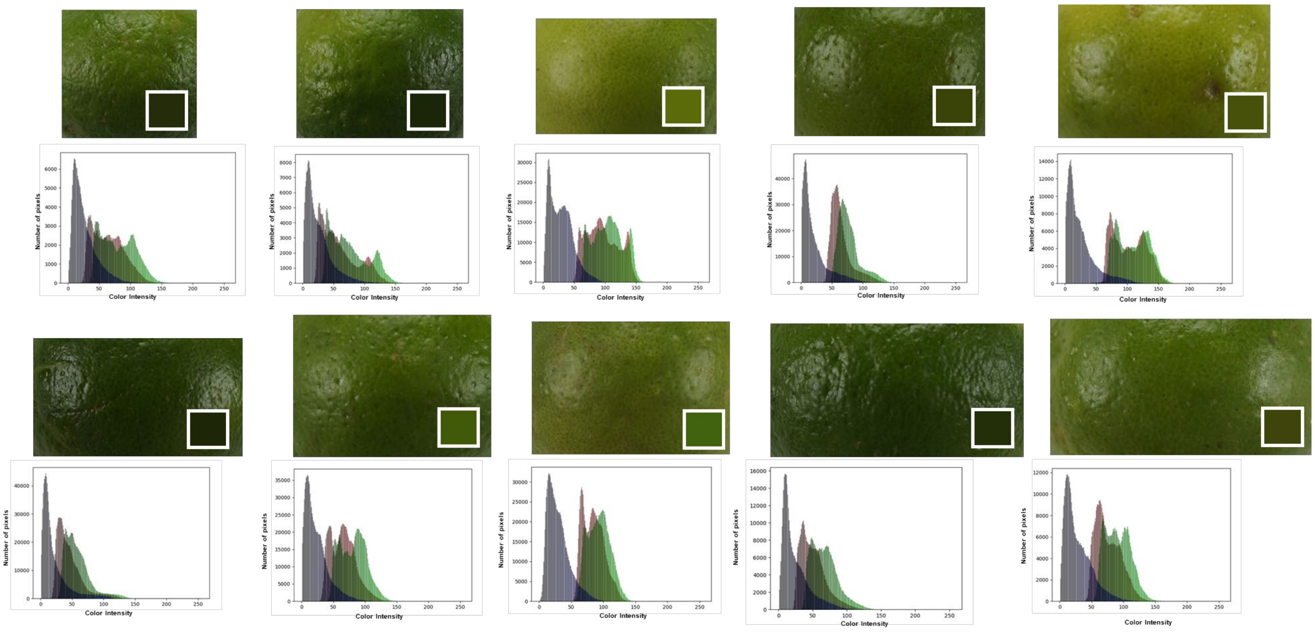

Supplementary Figure S1. Representative sample of 10 lemons analyzed for category "extra". Note: The differences in the dimensions of the images correspond to the condition of analyzing at least 60% of the total area of the fruit.

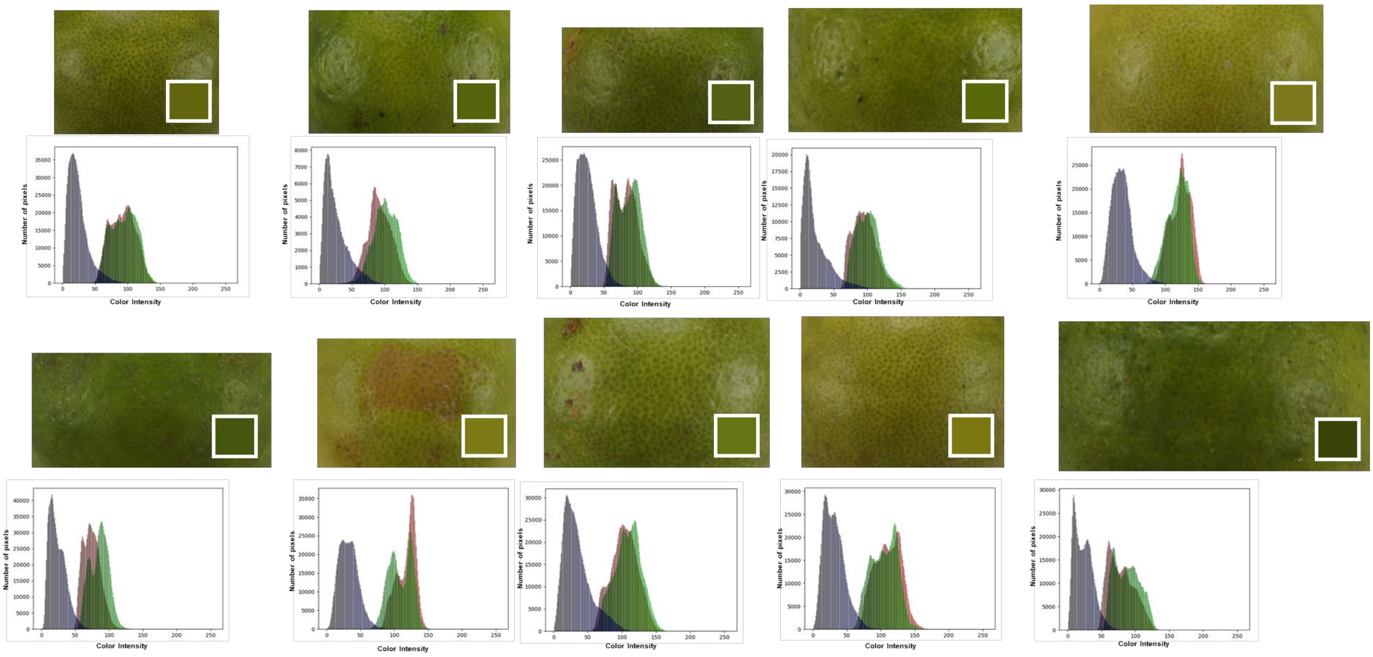

Supplementary Figure S2. Representative sample of 10 lemons analyzed for category I. Note: The differences in the dimensions of the images correspond to the condition of analyzing at least 60% of the total area of the fruit.

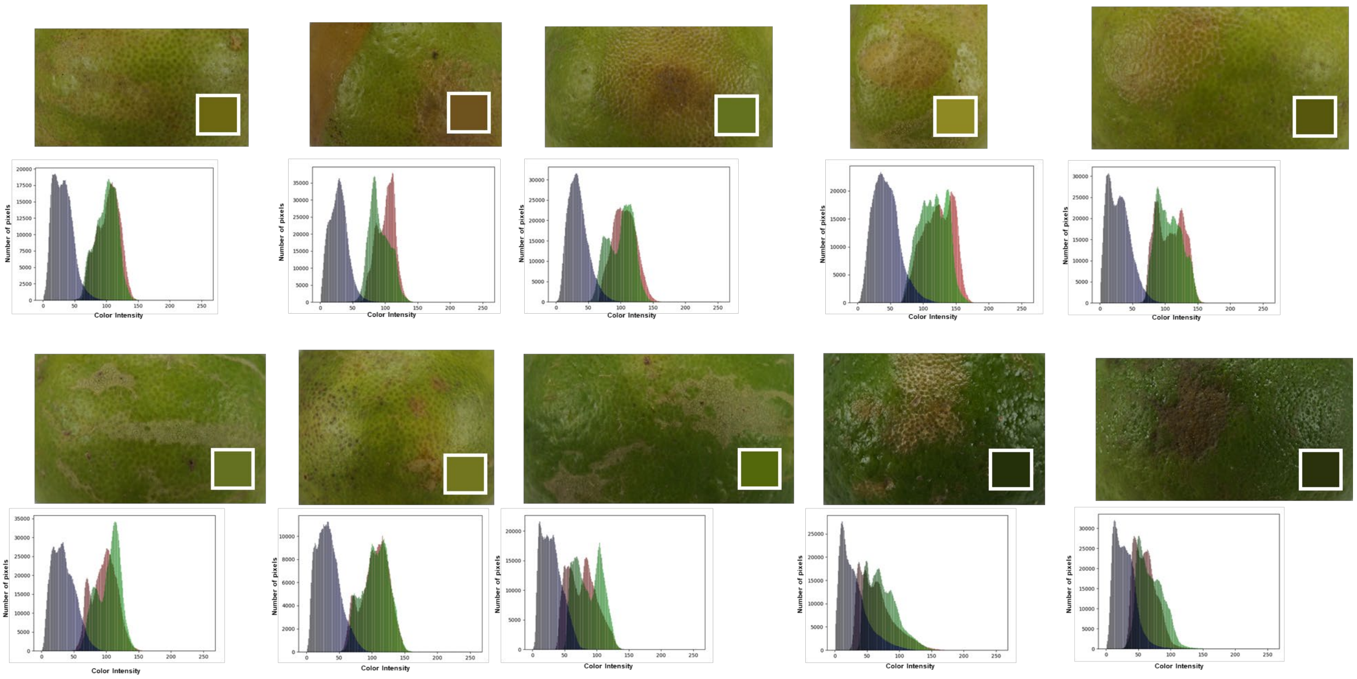

Supplementary Figure S3. Representative sample of 10 lemons analyzed for category II. Note: The differences in the dimensions of the images correspond to the condition of analyzing at least 60% of the total area of the fruit.

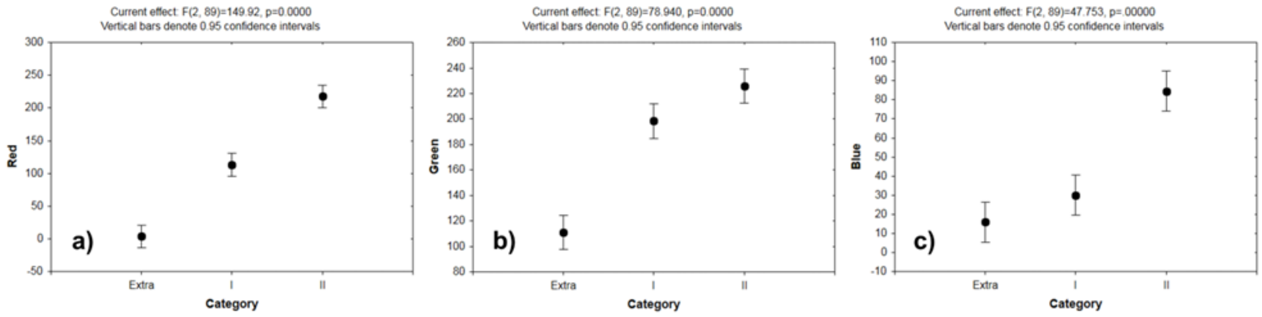

Supplementary Figure S4. Statistical analysis of the values of a) red, b) green, and c) blue in Persian lemons according to the category.

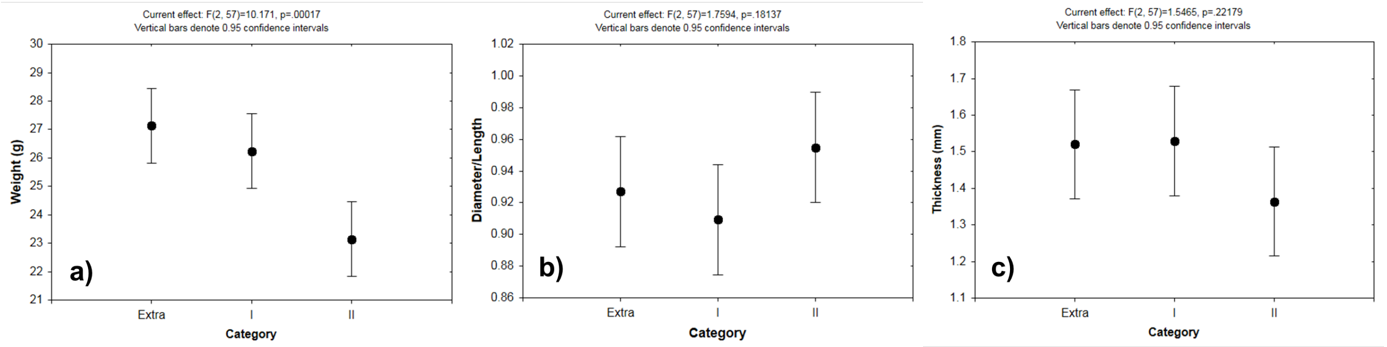

Supplementary Figure S5. Statistical analysis of the values of a) weight, b) diameter/length, and c) Thickness in Persian lemons according to the category.
